# Expert opinions on good practice in evaluation of health promotion and primary prevention measures related to children and adolescents in Germany

**DOI:** 10.1186/s12889-017-4773-y

**Published:** 2017-10-02

**Authors:** Katharina Korber, Christian Becker

**Affiliations:** 1 Munich School of Management and Munich Center of Health Sciences, Ludwig-Maximilians-Universität, 80539 Munich, Germany; 2 Institute for Health Economics and Health Care Management, Helmholtz Zentrum München (GmbH) – German Research Center for Environmental Health, 85764 Neuherberg, Germany

**Keywords:** Health promotion, Prevention, Evaluation, Expert interviews, Children and adolescents

## Abstract

**Background:**

Determining what constitutes “good practice” in the measurement of the costs and effects of health promotion and disease prevention measures is of particular importance. The aim of this paper was to gather expert knowledge on (economic) evaluations of health promotion and prevention measures for children and adolescents, especially on the practical importance, the determinants of project success, meaningful parameters for evaluations, and supporting factors, but also on problems in their implementation. This information is targeted at people responsible for the development of primary prevention or health promotion programs.

**Methods:**

Partially structured open interviews were conducted by two interviewers and transcribed, paraphrased, and summarized for further use. Eight experts took part in the interviews.

**Results:**

The interviewed experts saw evaluation as a useful tool to establish the effects of prevention programs, to inform program improvement and further development, and to provide arguments to decision making. The respondents’ thought that determinants of a program’s success were effectiveness with evidence of causality, cost benefit relation, target-group reach and sustainability. It was considered important that hard and soft factors were included in an evaluation; costs were mentioned only by one expert. According to the experts, obstacles to evaluation were lacking resources, additional labor requirements, and the evaluators’ unfamiliarity with a program’s contents. It was recommended to consider evaluation design before a program is launched, to co-operate with people involved in a program and to make use of existing structures.

**Conclusion:**

While in in this study only a partial view of expert knowledge is represented, it could show important points to consider when developing evaluations of prevention programs. By considering these points, researchers could further advance towards a more comprehensive approach of evaluation targeting measures in children and adolescents.

## Background

In recent years, health promotion and prevention programs have increasingly been implemented in Germany and also in other countries. Because of limited financial resources, only effective intervention measures should be adopted and, if possible, only the most cost effective [1]. However, the question of the costs and effects of health promotion and disease prevention measures has, until now, barely been answered, especially for children and adolescents and for settings in Germany. While there are many overviews on the effectiveness in the international literature, only some overviews of cost-effectiveness can be found for programs directed at adults [1, 2] or for distinct areas of health promotion and prevention, e.g., workplace-related programs [3–5].

Although health promotion and prevention measures are often directed at children and adolescents, even fewer attempts have been made to take into account the cost-effectiveness of health promotion and disease prevention measures for children and adolescents. Two recent systematic reviews exist on this topic (e.g. [6, 7]), which found only two economic evaluations for health promotion programs targeting children and adolescents in Germany [8, 9]. A much higher number of evaluations would have been expected, as for example, a relatively high number of prevention programs is listed in the “KNP-Projektdatenbank”, which includes all projects that were funded within the German interdisciplinary Prevention Research Funding Program, and thus gives an excellent overview of the German prevention landscape.

Given only few evaluations of primary prevention and health promotion measures that are directed at children and adolescents appear to be conducted, and in those that were conducted, by omitting cost consideration, an important aspect of evaluation appears to have been omitted, the question of good practice in evaluations of primary prevention and health promotion measures in children and adolescents arises.

Therefore, we aimed to explore expert opinions on good practice in measuring the costs and effects of health promotion and primary prevention measures and point out important implementation aspects of evaluations of health promotion and prevention measures for children and adolescents.

## Methods

Interviews were used to gather that expert knowledge and look for unknown obstacles to evaluation in programs involving children as these are hard to find in published studies [10, 11]. To itemize the aspects of implementation, the following items were incorporated in the interviews: the practical importance, the determinants of project success, meaningful parameters to be included in evaluations, supporting factors, and problems in the practical implementation of evaluations. With this overview, it is supposed to show already existing knowledge about the most important aspects of the implementation of (economic) evaluations and also to emphasize its importance for people responsible for the development of primary prevention or health promotion programs (for children and adolescents).

### Research approach

Expert interviews were chosen as the basic methodology [12]. As it seems appropriate in the context of this study that experts can report their own opinions on the subject, open interviews were conducted. So that the experts’ answers were a priori in a similar structure, the interviewers followed a guideline with possible questions (see section “Appendix”), from which they deviated if necessary (e.g., if the interviewee requested it). Therefore, a partially standardized structure for the expert interviews was chosen. As those interviewed were all from Germany, the interviews were held in German and the results are translated for this article.

The study specific definition of “expert” was made based on criteria by Meuser and Nagel [13]. In this study persons were considered as experts, if they were working in the area of health promotion/primary prevention (especially for children and/or adolescents). And additionally they should have a privileged access to information on the (economic) evaluation. This is also partly represented in question A2 of the guideline (Role of evaluation in daily work) and presented in the results section.

### Study process

Interviews were conducted and interpreted by the authors during July and August 2011. Both interviewers have a background of health economics and business research. The interviews had an average duration of approximately 35 min, ranging between 12 and 100 min in total.

We conducted a problem analysis based on a scoping review of published evaluations of prevention programs and documentation of existing prevention programs. Findings included that a large share of existing prevention programs directed at children and adolescents did not involve some type of evaluation (this could be seen based on the KNP-Projektdatenbank). Programs for which a scientific evaluation was published often addressed only a measure’s effects while costs were omitted. Following problem analysis, we developed an interview guideline that is described below. To achieve comparability of the answers, the guideline was designed to produce a high level of standardization. This means that the content and order of the questions were fixed. However, free formulation, reacting to requests, and ad hoc questions were possible. Such a semi-standardized procedure is considered suitable especially in areas where the expert serves as a source of information which otherwise would be difficult or even impossible to access; through free formulation, experts are given the possibility of introducing new aspects [13].

The guideline can generally be subdivided into the sections of probing questions (A) and general guideline questions (B–C) [14]. The probing questions, which were directed at assessing the respondents’ understanding of the term evaluation, the role evaluations had in their daily work, and at how they thought evaluations were perceived in practice, can be seen as introductory questions that helped the interviewers gain a more comprehensive view on the respondents and brought the respondents to the core questions of the interview in a comparable manner. The general guideline questions were aimed directly at aspects connected “good practice” and therefore addressed the study question. In particular, we asked questions about what were the benefits of conducting evaluations, what were measures for a program’s success, how this could be measured and on factors promoting or impeding evaluations.

### Participants

As prevention and health promotion projects are usually regionally anchored, people from the responsible regional institutions for prevention and health promotion were contacted. In total, 11 individuals from such institutions were contacted with a request for an interview. If the contacted individuals could not participate, they were asked to name a suitable replacement to be interviewed instead. In all interviews, two interviewers were present.

Eight respondents agreed to participate in the interview, which gives a participation rate of 73% (8/11). Two respondents were from public health insurance companies, four were from governmental and non-governmental research institutes, and further two were from local government.

Out of a total of eight interviews, four were conducted on-site in Munich, Germany. Another four interviews were conducted by telephone because the respondents were working in different parts of Germany.

All experts have academic backgrounds and they all were working in the area of health promotion and primary prevention or child and youth welfare.

### Evaluation strategy

The aim of the strategy is to emphasize supra-individual similarities, i.e. shared knowledge by comparing the texts of the interviews. The data analysis was made without the use of software and is based on an interpretative evaluation strategy for guided expert interviews [13, 15].

The procedure of the evaluation strategy is described below. The individual intermediate results of these steps are shown in the tables in this work.

The first step in the evaluation was the transcription of the acoustically recorded interviews. In the context of this study, nonverbal elements of the interviews were not taken into account. In order not to lose information and to avoid any corresponding distortion, a complete transcription of the recorded interviews was performed. This approach has the additional advantage that future studies with other objectives remain possible.

The second was paraphrasing of the individual interviews to condense the information available, thus reducing the complexity of the data. However, paraphrasing entails the danger of selective reproduction.

In a further step, the paraphrased parts of the interviews were assigned to topic-specific thematic headings. Comparable passages in the different interviews were identified and the corresponding headings were standardized.

A generalization is made by categorizing the headings created in the previous step that were in agreement across interviews. Within this step, the main statements of the experts were determined independently by two interviewers.

## Results

In the following passages, the major results are summarized and, at the end of the results section, an overview of the findings is given in Table 1. Detailed information on the questions and on the paraphrasing of the transcribed interviews by two independent interviewers can be found in Tables 2 and 3.

**Table 1:** Overview of expert responses to the interview questions

Questions	Summarized expert responses
A1. What does the term “evaluation” mean to you?	• Achievement of objectives • Assessment of measures • Evidence of effectiveness • Importance of systematic approach • Answer to the evaluation question • Way to optimize results and collect data • Naming of different evaluation types (process evaluation, outcome evaluation,…)
A2. What role does the evaluation of health promotion and prevention measures play in your daily work?	• (External) analysis of different evaluations • Support evaluations • Conduct evaluations on a defined framework for specific projects only as part of development of projects • Evaluation as main part of daily work
A3. Do you experience evaluations in practice as meaningful or rather disruptive? Please describe one example each for a meaningful evaluation or a less meaningful evaluation from your experience.	• Evaluation generally considered to be useful (for further development of programs, for supporting decisions such as financing) • Useful if specific given question is relevant • Useful if the measuring instruments are understood by participants and practitioners • No senseless evaluations • Evaluation can be considered as disruptive primarily by the “evaluated” • Evaluations can sometimes even be considered as a threat
B. What is your assessment of the practical importance of evaluation (in terms of measuring the costs and effects) of health promotion and prevention measures, especially in children and adolescents?	• Importance of evaluation rather indirect (information for public, politicians, funding institutions) • Evaluation can serve as a basis for and contribution to decision-making • Fulfills the need to examine costs in addition to effects/effectiveness • Evaluation can offer suggestions for further development of preventive work/preventive measures
C1. What aspects of health promotion and prevention measures (e.g., cost or effects) are particularly important to you, or when is a project successful for you? What would be an example of a particularly successful project in your opinion? (Why?)	• Project is successful when goals are reached, when actions are clearly attributable to effects • Effectiveness as a premise for offering a project, but different underlying criteria for effectiveness • Cost dimension (only mentioned by some experts) should be in reasonable proportion to effects • Projects should be transferable • Some examples of successful programs were given
C2. Which parameters would make such a success practically measurable?	• Sustainability of certain parameters depends on the specific aim of a prevention program • Distinction should be made between what was labelled hard factors (medical figures) and soft factors (i.e., lifestyle parameters or changes in physical activity) • Costs (only mentioned by one expert) • Considering risk factors and protective factors is seen as important • Longer follow-up periods for evaluation of results are seen as important • Representative studies needed to investigate acceptance, effectiveness, feasibility, and sustainability (not only for individuals but also for structures)
C3. What has proven to be particularly easy to implement regarding the implementation of the evaluation of health promotion and prevention measures in your experience?	• Good co-operation with all stakeholders to use their experience • Use of existing structures • Development of meaningful indicators for project success • Target group-specific design of the program • Evaluation concept should already be designed at the beginning of the measure
C4. What obstacles are to be expected in the evaluation of health promotion and prevention measures?	• Lack of acceptance by those affected (e.g., extra effort, no direct benefit, increasing documentation needs) • Missing “evaluation and quality culture” in practice • Evaluations often seen as a threat by those affected • Worries that results could be negative • Especially in the field of prevention, often only a few effects and no long-term evaluation • Evaluation of costs often problematic because of data protection matters (e.g., for health care cost data)

**Table 2:** Synopsis of the interviews transcribed, interviewer 1

Respondent	1	2	3	4	5	6	7	8
A1. What does the term “evaluation” mean to you?	°Verify achievement of objectives	°Evaluation as scientific proof of effectiveness	°Evaluation as systematic approach for the verification of achievement of objectives of projects. Supporting function of evaluation	°Measure effects and find statements regarding the cost-effectiveness relation	°Evaluation is survey of data to assess measures	°Evaluation as social scientific-based statement of the value or benefit of an item	°Evaluation as the assessment of the effectiveness of a measure	°Systematic collection of data in compliance with scientific requirements °Finally, an opinion regarding the project criteria should be made °Evaluation can refer to processes or results
A2. What role does the evaluation of health promotion and prevention measures play in your daily work?	°Performs evaluations itself (science)	°Sees evaluation as quality factor for programs that are offered or carried out itself	°Carries out evaluations itself °Orders evaluations. Co-develops the concept	°Active in development of prevention offers and their evaluation	°Carries out evaluations of prevention projects itself	°Carry out evaluation projects themselves	°Works at research institute, which evaluates prevention measures itself	°Edits project proposals and final reports °Passes recommendations for improvements
A3. Do you experience evaluations to be perceived in practice as meaningful or more disruptive? Please describe one example each of a meaningful evaluation or a less meaningful evaluation from your experience.	°Evaluations are useful if °useful questions/ target criteria exist °effects are relevant for intervention and interest in knowledge is present °suggestions for further development are given °comprehension of practitioners takes place °practitioners understand instruments	°Useful as decision support for financing °Is evaluation for cash useful at all? (cost savings desired. Cannot be verified in monetary terms) °Disturbing regarding the detection of “soft factors” (if outcomes are not easily measurable) °Less useful if there are no meaningful target criteria	°Dependent on questioning °Can be experienced as an extra burden	°Disturbing because of great effort	°Too expensive if every single small project is evaluated	°Practitioners might feel uncomfortable with evaluations if they are examined carefully and their routines are broken	°Is possibly seen as disturbing by the prevention experts in the field, not so much on the part of the target groups °Evaluation from supplier’s point of view is expensive and costly °Abstract results from journals do not have a reference to the practitioner’s living environment °Institutions could see themselves as “cash cows”	°Can be experienced as unnecessary °In practice also partly fear of the evaluation due to lacking knowledge °Evaluation can also be experienced as a threat °From a scientific point of view, evaluation offers chances
B. What according to your assessment is the practical importance of evaluation (in terms of measuring the costs and effects) of health promotion and prevention measures, especially in children and adolescents?	°Scientific view can give impulses °Evaluation cannot clarify whether the program makes sense °Contributes to further development of measures (based on ideas of practice), if desired effects are unpresentable °Evaluation is important for the public, politicians, and investors °Practical importance given if not only PR-effective factors are examined	°Practically relevant if a long-term follow-up exists	°Evaluation as basis for decision-making °Costs as argument °Soft variables possibly not helpful for political decisions	°Evaluation gives hints on what has an effect and what has no effect	°Important to deliver projects to be good practice °Evaluation gives suggestions to improve preventive work/measures °Especially regarding very innovative, cost-intensive projects, evaluation should take place °Evaluation discloses new aspects °Evaluation as enlargement of the people involved (new point of view)	°Evaluations open new perspectives for the people involved °Evaluations address specialists and the structures they work in °Target individuals can be involved by participatory forms	°No direct practical meaning as measures are also offered without evaluation. On a meta level, however, evaluations are a condition for implementation °Evaluation to choose between different measures	°Evaluation important °Cost analysis helps to convince the decision-maker and to continue a measure
Please describe, based on your experience, one example each for a useful evaluation or a less useful evaluation.	°Useful: Life-competence projects in the prevention of drug dependence/Tiger-Kids^a^	°Safari-Kids^b^	°“Münchner Modell der frühen Hilfen”^c^	°Absence time report, workplace health promotion				°Participative approach of movement promotion for underprivileged women
C1. What aspects of health promotion and prention measures (e.g., cost or effects) are particularly important to you, or when is a project successful for you? What would be an example of a particularly successful project in your opinion? (Why?)	°Achievement of objectives (to influence risk and protection factors) °Reaching the target group °Transfer °Acceptance of the performing person °Qualified target group °Sustainability °Broad effectiveness	°Feasibility, participation, and sustainability	°Successful if people are addressed who benefit most from it	°Good effectiveness with appropriate cost employment °View of the concerned individuals shall be considered °Evaluation shall not only reach the middle class	°Participation (can reduce costs) °Participative development of quality °It is also a success if only a few are helped °Comparability °Textual consideration	°Connection between taking an action and the effects must be clearly produced °Comparison with initial state must take place °If subjective points of view of the people concerned are included in the evaluation and feedback is obtained from those people °Considering also the benefit of the principal °If the measure is effective	°Primary effects °Costs also important °Broad effectiveness important °Premise is that a program has an effect. Program should be realizable in terms of costs °Validity is important: does the project produce what it expected to cause? °Important if target group is achieved °Successful project should have concrete questioning, should be able to be broadly used, or should address special target groups purposefully	°If targets are achieved °If achievement of objectives can be attributed to the measure °Has to be feasible under real conditions °Sustainability regarding effects and transferability °Must not be offered only once as a project
C2. Which parameters would make such success practically measurable?	°Protective factors as important as target figure °Risk and protective factors with scales °Representative studies	°There are hard and soft factors; however, in the purely preventive area, the soft factors are more important (growth, etc.)	°By observation of acceptance, availability	°Investigation of long-term stability of behavior °Sustainability of behavior and structures	°Reachability of participants °Achievement of objectives °Costs	°Dependence of object and operationalizability °Can be soft or hard parameters	°Regarding validity, lifestyle parameters would be interesting or physical activity °Measurements before and afterwards and longer follow-up important °Protocol, what is implemented, when and where would be important	°Effectiveness depends on the individual case °Sustainable implementation can be proven by documentation °For the transfer, it is important to create transfer aids
C3. What has proven to be particularly easy to implement regarding the implementation of the evaluation of health promotion and prevention measures in your experience?	°Work closely together with those performing the task and introduce their expertise	°Address family/environment (sustainability) °Use settings (existing structures)	°Adapt the survey tools to target groups	°Keep effort low	°Tight communication is most helpful for the project	°Stakeholder participation and transparency	°It is helpful to use existing structures rather than to approach individuals	°Plan evaluation from the beginning °Minimal requirements that always work: intervention group, control group (not absolutely randomized) °Consider useful indicators
C4. What obstacles are to be expected in the evaluation of health promotion and prevention measures?	°Time °Staff °Motivation °Institutions in practice overstrained by evidence base °Motivation of participants as part of evaluation	°Great effort °Understanding of the people involved °No clear causality of the measure recognizable °Cost involvement difficult due to privacy, especially when no manifest disease °Costs that are influential as a result of prevention are not directly recognizable °Consideration of costs only useful with long-term observation °Effects only recognizable after a long time	°External evaluation of those possibly not familiar enough with the project °Participation is target group dependent and cultural differences have to be noticed	°Too much effort is an obstacle °Lacking acceptance by those concerned	°Barrier if evaluation is performed during current operations instead of already planned before °Evaluation is felt as being irksome	°Wrong information °Refusal °Those concerned do not gain any benefit °Objective is adapted by principal °Those concerned do not want to be examined precisely °Evaluation abused as delay in decision-making °Different interests, mistrust (scare sponsors off) °Consent of parents for comprehension of children	°Recruitment of those who one wants to reach is difficult (prevention bias) °Difficulties in determining whether effects also appear outside laboratory conditions (efficiency vs. efficacy) °Long-term follow-up	°In children, effects appear time-lagged °Effects of evaluations are never very strong °Objectives are never reached at all °Missing evaluation and quality culture °Evaluation is felt as a threat °Measures are complex °Cost aspects °Control group does not see any benefit from participation

^a^TigerKids: Strauss A, Herbert B, Mitschek C, Duvinage K, Koletzko B. [TigerKids. Successful health promotion in preschool settings]. Bundesgesundheitsblatt Gesundheitsforschung Gesundheitsschutz. 2011 Mar;54(3):322-9

^b^Safari-Kids: https://www.dak.de/dak/leistungen/SAFARIKIDS-1079158.html

^c^Münchner Modell der frühen Hilfen: https://www.muenchen.de/rathaus/Stadtverwaltung/Sozialreferat/Jugendamt/Familie/Fruehe-Hilfen.html

**Table 3:** Synopsis of the interviews transcribed, interviewer 2

Respondent	1	2	3	4	5	6	7	8
A1. What does the term “evaluation” mean to you?	To verify whether targets aspired to were reached ➔ achievement of objectives	Proof of efficacy, proof that positive results emerge, arrangement of evaluation not defined	Approach or process for the evaluation of measures, important: systematic approach, systematic review of achievement of objectives. Evaluation of endpoints as well as process evaluation is important to optimize results	Measure effects, but also learn something about cost–benefit relation	Collection of data or findings for the evaluation of a measure	Socio-scientific-based assessment of value or benefit of an object; object can be program, institution, structure, does it work, and are objectives achieved? ➔ achievement of objectives	Evaluation but also the question after the effect of a measure	Collection of data and analyzing an object. Important here: systematic implementation has to satisfy scientific requirements. Evaluation should be included so that position statement also possible, questioning of the evaluation has to be answered
A2. What role does the evaluation of health promotion and prevention measures play in your daily work?	Majority of work ➔ main business, involved in development, implementation, and interpretation	For assessment of programs always a factor, but itself not involved in evaluation, only accompanying action	Important role, main task, partly evaluations self-designed, implemented, and analyzed, partly main business, involved in development, accompanies implementation and analysis	Important role, because while developing new prevention programs, evaluation is also developed immediately	Evaluation broad: Analysis of own work: constantly, evaluation closely: after a fix frame for specific projects ➔ sideline, focus on projects, evaluation as additive	Quietly business field, everyday business, evaluation of federal programs, legislative initiative, practical approaches➔main business, involved in development, implementation and analysis	Main business, involved in development and analysis	Not involved in development, implementation, and analysis itself but everyday business is the assessment of programs and projects, where evaluations should or must also be included
A3. Do you experience evaluations to be perceived in practice as meaningful or more disruptive? Please describe one example each of a meaningful evaluation or a less meaningful evaluation from your experience.	Basically useful, if you have a specific question and if it serves for further development, clear target criteria less useful: measuring instruments which are not understood by those people who should apply them	Both, useful for investor, disturbing for program suppliers, implementers, involved persons; example: Safari-Kids	Useful vs. disturbing does not exclude each other, can be useful and nevertheless disturb ➔ if evaluation is useful or not also depends on whether the data are used sensibly or not, e.g., early aids	In practice, often sensed as disturbing due to effort, e.g., AOK-absence-time report	Useful if prior defined criteria are checked; problematic or disturbing for the peoples involved because of high effort ➔ depends on what is done and who is affected	Partly useful, but aware that one possibly disturbs the “evaluated” ➔ more useful Example (less useful): program evaluated, objectives not reached, nevertheless no change and vice versa, program evaluated, very good results, nevertheless program terminated ➔whether evaluation useful or not also depends on whether data are used in a proper way or not	Possibly sensed as disturbing or can sometimes be sensed as disturbing by people or prevention workers who work in the field and do not immediately get in touch with the results. There are no useless evaluations; if ever there are bad evaluations	Partly useful, but from experience, evaluations are often seen as useless in practice, possibly even as a threat. Most important insight from the examples: clear objective, which one wants to reach and is verifiable; this often goes wrong
B. What according to your assessment is the practical importance of evaluation (in terms of measuring the costs and effects) of health promotion and prevention measures, especially in children and adolescents?	Scientific view on something that is done in everyday life in practice and can give impulses. Not: this makes sense or not, but an impulse for further development of the measures, therefore may have practical importance but not the non plus ultra	Useful if long-term observation is included	Basically important for decision-making, therefore a cost–benefit analysis would also increase importance	It is important to make sure what has an effect and what does not, especially regarding children and adolescents; evaluations are important to find out what has an effect or what is useful and what is not	Important: quality of the measure, thus quality in planning and implementation has to be present and has to be improved; projects get more and more costly and bigger, here an involvement of evaluation is important	Evaluations are initially not directly addressed to children and adolescents, but rather to specialists and structures in which children move, i.e., school. Implementation of evaluation results or possible recommendations also always dependent on how this fits into existing structures. The closer one is to the decision-makers, the more useful and momentous evaluations can be for children and adolescents. If one involves children and adolescents directly in the evaluation (with different perspectives), then they have an opportunity to take part in the design	Of course one can also implement prevention measures completely without evaluation; thus, it has no immediate practical importance. On a meta level, I would say, however, that it actually is a condition for implementation in practice or at least should be one	Extremely important, especially regarding the costs; decision-makers can possibly be convinced better if one can give information about costs as well as effectiveness
Please describe, based on your experience, one example each for a useful evaluation or a less useful evaluation.	°Useful: Life-competence projects in the prevention of drug dependence/Tiger-Kids^a^	°Safari-Kids^b^	°“Münchner Modell der frühen Hilfen”^c^	°Absence time report, workplace health promotion				°Participative approach of movement promotion for underprivileged women
C1. What aspects of health promotion and prevention measures (e.g., costs or effects) are particularly important to you, or when is a project successful for you? What would be an example of a particularly successful project in your opinion? (Why?)	Project is successful if objectives are achieved and especially the planned target group is achieved. Transferability. Successful prevention influences risk factors and protection factors, feasibility, acceptance, sustainability, example: Tiger-Kids	Feasibility, participation, sustainability, example: Safari-Kids	Overall very general, always dependent on the question, but for us: accessibility of target group (disadvantaged people)	Successful would mean a good effect with appropriate cost deployment, dimension of effects most important, costs have to remain within reasonable bounds, participation of the people involved, i.e., participative development of quality, reference to absence report, operational health promotion as best developed approach to prevention	Participative development of quality, thus involvement of the people affected, evaluation may not change the project, fixed criteria which should be comparable, overridingly assessment concerning the contents of the success, thus with regard to the objectives of the project not of the evaluation	Connection between action and effect ➔ imputability, separation of prevention in the narrow sense and health promotion, different approaches, different prerequisites for evaluation, who uses the results? Participative design whenever possible	Premise for offering a program at all is that is has an effect. Simultaneously, it has to enable a realistic feasibility in terms of costs. Validity, attainment of the target group, achievement of objectives	Feasibility, sustainability, transferability, for example a project of movement promotion for disadvantaged women, which meets these criteria; however no dramatic effects established, only small changes
C2. Which parameters would make such a success practically measurable?	Effectiveness, attainment of target groups, feasibility, sustainability, to measure with appropriate representative studies	Hard factors (weight, blood values,…) and soft factors (fun); in children, more soft ones, at least in the preventive area	Acceptance of target group as a parameter of accessibility	Individual: behavior; institution: to which extent are sanitary aspects, sanitary perspectives in processes and structures taken into account	Accessibility, costs, achievement of objectives	Always dependent on project and cognitive interest	Dependent on target, this can be so called lifestyle parameters, on the one hand, but also recording	Always dependent on project, but finally one can achieve much by means of documentation
C3. What has proven to be particularly easy to implement regarding the implementation of the evaluation of health promotion and prevention measures in your experience?	Working at eye level with those involved, creating a win–win situation, also considering the practitioner’s “gut feeling”, but not only	Considering the whole environment, using networks, going into the settings	Dependent on the measure, looking at exactly which instrument is most suitable for which target group	Low effort	Close communication with those involved, identification with the project target	“Assessment of evaluability”, involvement of all people concerned, transparency, and clearly defined targets	Using the structures given	Planning evaluation from the beginning, good definition of objectives, intervention group, defined and large enough, ideally also control group
C4. What obstacles are to be expected in the evaluation of health promotion and prevention measures?	Time, staff, motivation, oversaturation of the practice with all kinds of evaluations from different areas, communication of the effects	Effort, costs, financial and personnel resources, understanding of the people involved	Money, appropriate contractor, familiarity with the object to be evaluated, linguistic shyness to engage oneself in something written, generally finding the access	High effort	Lack of resources, effort is often felt as stress	Refusal to participate, wrong information, wrong data, boycott in every possible form, balances of power, privacy, mistrust	The one obstacle is the recruitment, worse accessibility of specific groups of people, another obstacle in evaluation is that it is relatively difficult to create such real-life evaluations, thus one distinguishes sometimes between so called efficacy and efficiency evaluation; therefore, I can actually point out on the basis of the results, which I get in such a study, that this will then have those effects in reality	Lacking quality culture of evaluation, that evaluation is sometimes felt as a threat and not as a chance, lacking competencies and capacities, methodical difficulties, money, … Participants do not want to take part, some measures are “bound politically to succeed”

^a^TigerKids: Strauss A, Herbert B, Mitschek C, Duvinage K, Koletzko B. [TigerKids. Successful health promotion in preschool settings]. Bundesgesundheitsblatt Gesundheitsforschung Gesundheitsschutz. 2011 Mar;54(3):322-9

^b^Safari-Kids: https://www.dak.de/dak/leistungen/SAFARIKIDS-1079158.html

^c^Münchner Modell der frühen Hilfen: https://www.muenchen.de/rathaus/Stadtverwaltung/Sozialreferat/Jugendamt/Familie/Fruehe-Hilfen.html

### Introductory questions and questions regarding the background of the experts

#### A1. Meaning of the term “evaluation”

Overall, the respondents had a relatively similar basic understanding of evaluation that encompassed the review of achievement of objectives, evaluation or assessment of measures, and evidence of effectiveness. Notably, about half of the respondents specifically addressed the systematic or scientific approach underlying evaluation, which also includes defining evaluation objectives. Several respondents referred to specific types of evaluation, in particular, process evaluation, endpoint evaluation, outcome evaluation, or cost–benefit ratio were mentioned. Further views on evaluation were that it was as a way to optimize results, to collect data, and to take a stand for a project based on the respective results.

#### A2. Role of evaluation in daily work

For the majority of respondents evaluation represented the main component of their daily work. Five individuals were directly involved in project development, implementation, and evaluation, each with a different focus. One expert had a supportive function and one respondent’s occupation was the development of prevention projects, whereas the evaluation was a secondary focus. Finally, one of the experts interviewed was not involved in the development, implementation, and analysis of evaluations, but rather in the assessment of programs and project proposals.

#### A3. Personal perception of evaluation in practice

As it turned out in the course of contacting the experts that no expert from “practice”, that is, none is confronted with both the execution and the implementation of a program and its evaluation, this question was expanded to also include a personal view on how evaluations are perceived in practice.

As described above, all respondents generally consider evaluations to be perceived as useful: on the one hand, for further development of programs and, on the other hand, for supporting decision making, for example with regard to financing. Furthermore, the respondents found it to be important that the measuring instruments be understood by participants and practitioners, who should be involved in the development already. One of the respondents stated that there are no senseless evaluations in the proper meaning, and if at all, only badly designed ones.

Shared opinion among respondents was that “meaningful” and “disruptive” were not mutually exclusive. They all reported from their experience that evaluations could be considered to be disruptive and definitively were, primarily by the “evaluated”, i.e., those who are directly involved (for example, program providers or participants). In addition, there was agreement that in practice disruption was experienced predominantly because evaluations were always associated with extra time. Some of the respondents indicated that evaluation can in practice be considered a threat in that routines could be broken or there could be fear that a program could turn out not to be as successful as anticipated.

### Significance of evaluation

In part B of the interview, the experts were asked to give an assessment on what makes an evaluation important in practice.

Most experts agreed on the relevance evaluation to decision-making. In particular, it was stated that an evaluation provides insights on a measure’s effect and generates data to inform program improvement and further development. Thereby, as three experts noted, the scientific approach involved in evaluations could open up new perspectives to those involved and offers an interface for involvement of patients and providers.

Hard outcomes and results of evaluations with long-term follow-up were seen especially relevant. Moreover, information on the measures’ costs was seen to be particularly important as an argument with regard to a measure’s implementation or justification. Beyond reaching decision makers, communicating evaluation results was seen as a public relations channel with further audiences, such as investors or the public in general.

Additionally, some examples of what the respondents thought were successful preventive programs were named (see in Table 1).

### Evaluation methods

#### C1. Determinants of prevention project success

In this part of the interview, the experts were asked to give a subjective assessment of when a prevention project is successful and which aspects they consider to be particularly important.

To the majority of respondents, an important determinant of a prevention program’s success is that a prevention program is effective and that causality is visible. Effectiveness has been stated to be a premise that the project is offered at all. While some respondents would call a measure successful even if its effectiveness was minimal, several others also mentioned the cost dimension, which should be in reasonable proportion to the effects. Aside from effectiveness in terms of addressing, for example, risk and protective factors, several experts pointed out that is was particularly important that a prevention program reaches its desired target group. For instance, as one respondent noted, a program should reach those people who benefit most from it and not just the interested middle class. Most respondents agree to that. In addition, it was emphasized the measure should be accepted by the people providing it and actively involve the participants. In this connection, a process of participatory quality improvement through feedback was pointed out.

Moreover, the respondents considered those projects to be successful that are transferable, i.e., that could be implemented under real conditions and were sustainable beyond the end of the project.

#### C2. Parameters desirable for inclusion in an evaluation

In this section of the interviews, the experts were asked to name parameters, which they thought would allow measuring a prevention project’s success.

Several experts pointed out that documentation and valid study designs were an important factor in making a measure’s success measurable. With regard to which factors to observe, a number of experts emphasized that these would essentially depend on the individual case. Some experts pointed out the general distinction of what they referred to as “hard factors such as medical figures” and “soft factors such as lifestyle parameters or physical activity”. Costs were mentioned by only one person. Overall, the majority of experts considered less objectively quantifiable factors (such as “acceptance” of the program or “fun” for example) as suitable for assessing the success of measures in the purely preventive field. In addition to risk factors, protective factors were seen important. Both, however, should be examined after a longer follow-up period to examine long-term stabilization of behavior and sustainability of structures.

#### C3. Factors supporting evaluation

The experts were asked to name easy-to-implement evaluation approaches.

Several experts noted that an evaluation concept should be designed at the beginning of a measure to support subsequent evaluation. It was seen important to co-operate closely with those performing the work (all stakeholders) and to bring in their expertise. Furthermore, it was found to be helpful to make use of existing structures (settings). In terms of sustainability, however, the family or environment should also be addressed. Finally, using existing survey tools that were adapted to the intended audiences were mentioned.

#### C4. Obstacles to evaluations

In this section, respondents were asked to provide information about possible obstacles to the evaluation.

Most often, a lack of resources and acceptance by those affected was mentioned, which included participants, control subjects, and providers of preventive measures. The experts stressed that an evaluation means a lot of extra effort for those affected. In particular, institutions were seen as overburdened by the increasing documentation needs involved with evidence-based analysis and were not likely to express sympathy toward evaluation. Here, the lack of evaluation and quality culture in practice was criticized. Furthermore, evaluation were stated to be frequently seen as a threat by those affected, who did not want to be examined carefully, and institutions, who could worry that their measure would not receive funding anymore because of the evaluation results.

Another important point raised by the experts relates to the effectiveness of the measure. Regarding external evaluators, it was seen primarily necessary to familiarize them sufficiently with the project. If no clear causality mechanism was recognizable an evaluation would likely not capture the program’s intended effects. Thereby, it was noted that particularly for complex measures effects observed in evaluations were often not particularly strong. According to the experts, this casted doubt on whether effects would also show outside the laboratory conditions. Long-term follow-ups were seen unlikely to be feasible, but effects would often only be seen after a long time, especially regarding prevention projects addressing children.

Finally, the aspect of cost involvement was addressed. Obtaining data on the participant’s health expenditure is seen to be difficult owing to data protection. Costs that can be influenced by prevention were also not apparent without long-term observation.

## Discussion

In this study, eight experts were interviewed on topics in evaluations of prevention programs addressing children and adolescents, which include practical importance of evaluations, determinants of project success, parameters desirable to be included in evaluations, supporting factors, and also on problems in the implementation of evaluations in practice.

Because of the relatively small number interview participants it can be assumed that only a part of the expert knowledge can be represented. However, even within this small sample, we observed what appeared to be theoretical saturation regarding some questions, which means that no additional findings could be gained. A drawback is that among the non-responders and those who refused to participate in the interview were people who were likely to have experience of being affected by an evaluation (that is, as someone who is “evaluated”). This is consistent with the assessment by the interviewed experts that people affected by evaluation might consider evaluations, and possibly also the present investigation, to be complex without recognizing the underlying purpose. Thus, the inclusion of this perspective, unfortunately, was not immediately possible, but is targeted for more far-reaching surveys. Owing to the small sample and the willingness to participate, a special selection of participants could have resulted. It can be assumed that those who gave positive feedback regarding the participation have a positive attitude toward the evaluation of programs.

The experts indicated that evaluations had a practical relevance, among others, because these generated arguments for decision makers. Accordingly, several experts found it to be an important determinant of prevention program success that costs should be in reasonable proportion to effects. In contrast, only one expert thought of costs an important parameter to include in an evaluation. This was surprising, as costs represent a higher level aspect that equally applies even to heterogeneous measures. However, it appears that there is an awareness of the relevance of economic aspects in evaluations, but still its implementation is problematic, even for experts.

One possible explanation of why costs might not be considered so much in evaluations of prevention programs addressing children and adolescents could lie in the nature of primary prevention, which seeks to avoid disease before it manifests itself. Within the often short time horizons of such evaluations it can be assumed that the supply side immediately faces costs (e.g., for the implementation of the action), whereas monetary benefits of prevention, which mainly consist of avoided expenditures in the distant future, could not be observed [11]. The consideration of costs for prevention programs for children and adolescents therefore puts a disadvantage on these offers initially. This is of great importance, as the evaluation is seen primarily as a foundation for decision-makers. This issue is also reflected by the literature. Although there are several more or less detailed guides how to implement economic evaluations [16–19] there is still need on more practical oriented tools to reach higher acceptance of economic evaluation also by practitioners [11, 20]. First steps in this direction have already been taken [21].

From the experts’ statements it appeared that compared to costs a prevention program’s health effects were considered to be more important. However, measuring health-specific effects for purely preventive measures in healthy children and adolescents can also be problematic. Especially in health promotion programs directed at children, which often address healthy populations, health effects cannot usually be quantified in terms of patient relevant outcomes or even medical markers that can be measured objectively. Instead, less objectively quantifiable effects are focused on, including a wide variety of measures of physical activity. In this context, fuzziness with regard to how to measure such effects and a low comparability across studies are certainly fields of improvement. In addition, such effects cannot be easily translated into patient-relevant outcomes beyond the observation period for example in context of health economic modelling. The inclusion of prevented outcomes would require assumptions about a relationship of intermediate outcomes and final outcomes and the future development, which is subject to a high degree of uncertainty. Thus, several experts pointed out that the effect of preventive measures is often overestimated. A new vision for what prevention can achieve would have to be developed. Thereby, close cooperation between evaluators and people in the setting would appear to be helpful. Perhaps, this new perspective would reduce reservations with respect to evaluations in the evaluated and lead to more productive field work.

## Conclusions

For the reasons mentioned above, it could be useful to understand prevention as a comprehensive approach and to include this in the evaluation. Instead of constantly evaluating many small projects in terms of costs and effects, the evaluation should be performed as an ongoing cross-sectional task (similar to quality assurance). Thereby, it would be important to also consider factors, such as outreach to target groups, or psychosocial determinants, which draw attention to long-term behavioral changes (in terms of sustainability).

In conclusion, by conducting expert interviews we obtained insights into practitioners’ views on “good practice” in evaluation of health promotion and disease prevention measures. Important components of evaluations of prevention measures were pointed out and the need for research regarding development and advancement, especially in terms of economic aspects, was made very clear. This article also emphasizes the importance for stakeholders involved in developing or running prevention or health promotion programs to retaining the economic aspect of evaluation, which can provide important arguments for such programs in light of limited financial resources.

## Appendix

Guideline:

(Economic) evaluation of health promotion and prevention measures related to children and adolescents.

A1. What does the term “evaluation” mean to you?

A2. What role does the evaluation of health promotion and prevention measures play in your daily work?

A3. Do you experience evaluations to be perceived in practice as meaningful or rather disruptive? Please describe one example each for a meaningful evaluation or a less meaningful evaluation from your experience.

B. What according to your assessment is the practical importance of evaluation (in terms of measuring the costs and effects) of health promotion and prevention measures, especially in children and adolescents?

(Please describe, based on your experience, one example each for a useful evaluation or a less useful evaluation.)

C1. What aspects of health promotion and prevention measures (e.g., cost or effects) are particularly important to you, or when is a project successful for you? What would be an example of a particularly successful project in your opinion? (Why?)

C2. Which parameters would make such a success practically measurable?

C3. What has proven to be particularly easy to implement regarding the implementation of the evaluation of health promotion and prevention measures in your experience?

C4. What obstacles are to be expected in the evaluation of health promotion and prevention measures?

## Abbreviations

DFG: Deutsche Forschungsgemeinschaft (Organisation for Science and Research in Germany)
KNP-Projektdatenbank: Database of the “Kooperation für nachhaltige Präventionsforschung” (Cooperation for sustainable prevention research)
